# Suppression of the JAK/STAT Pathway Inhibits Neuroinflammation in the Line 61-PFF Mouse Model of Parkinson’s Disease

**DOI:** 10.21203/rs.3.rs-4307273/v1

**Published:** 2024-05-07

**Authors:** Huixian Hong, Yong Wang, Marissa Menard, Jessica Buckley, Lianna Zhou, Laura Volpicelli-Daley, David Standaert, Hongwei Qin, Etty Benveniste

**Affiliations:** University of Alabama at Birmingham

**Keywords:** Parkinson’s Disease, Neuroinflammation, α-Synuclein, JAK/STAT, AZD1480, scRNA-Seq

## Abstract

Parkinson’s disease (PD) is characterized by neuroinflammation, progressive loss of dopaminergic neurons, and accumulation of a-synuclein (a-Syn) into insoluble aggregates called Lewy pathology. The Line 61 a-Syn mouse is an established preclinical model of PD; Thy-1 is used to promote human a-Syn expression, and features of sporadic PD develop at 9–18 months of age. To accelerate the PD phenotypes, we injected sonicated human a-Syn preformed fibrils (PFFs) into the striatum, which produced phospho-Syn (p-a-Syn) inclusions in the substantia nigra pars compacta and significantly increased MHC Class II-positive immune cells. Additionally, there was enhanced infiltration and activation of innate and adaptive immune cells in the midbrain. We then used this new model, Line 61-PFF, to investigate the effect of inhibiting the JAK/STAT signaling pathway, which is critical for regulation of innate and adaptive immune responses. After administration of the JAK1/2 inhibitor AZD1480, immunofluorescence staining showed a significant decrease in p-a-Syn inclusions and MHC Class II expression. Flow cytometry showed reduced infiltration of CD4^+^ T-cells, CD8^+^ T-cells, CD19^+^ B-cells, dendritic cells, macrophages, and endogenous microglia into the midbrain. Importantly, single-cell RNA-Sequencing analysis of CD45^+^ cells from the midbrain identified 9 microglia clusters, 5 monocyte/macrophage (MM) clusters, and 5 T-cell (T) clusters, in which potentially pathogenic MM4 and T3 clusters were associated with neuroinflammatory responses in Line 61-PFF mice. AZD1480 treatment reduced cell numbers and cluster-specific expression of the antigen-presentation genes *H2-Eb1, H2-Aa, H2-Ab1,* and *Cd74* in the MM4 cluster and proinflammatory genes such as *Tnf, Il1b, C1qa,* and *C1qc* in the T3 cluster. Together, these results indicate that inhibiting the JAK/STAT pathway suppresses the activation and infiltration of innate and adaptive cells, reducing neuroinflammation in the Line 61-PFF mouse model.

## INTRODUCTION

Parkinson’s disease (PD) is the most common motor neurodegenerative disorder, affecting 2–3% of the population over 65 years of age (1, 2). Immune system dysfunction, genetic mutations, and environmental factors contribute to its pathogenesis (3), while polymorphisms in the HLA-DR (MHC Class II) locus increase risk of its development (4). Studies show that anti-tumor necrosis factor (TNF) and nonsteroidal anti-inflammatory treatments are associated with a reduced incidence of PD symptoms (5–7).

The core pathological features of PD are neuroinflammation (8), a loss of dopamine-producing neurons, and the accumulation of misfolded, aggregated α-synuclein (α-Syn) in the substantia nigra pars compacta (SNpc), called Lewy pathology (1). In postmortem tissue, Lewy pathology is associated with enhanced MHC Class II expression on microglia and infiltration of macrophages and T-cells in the midbrain (9–11). Neuroimaging studies confirm chronic myeloid activation in the brains of PD patients (12), and increased numbers of α-Syn-reactive T-cells circulating in the blood of PD patients demonstrate immune cell activation (13–15).

Research has focused on the neuroinflammatory features of PD, particularly endogenous microglia (MG) and monocytes/macrophages (MM), which infiltrate from the periphery (3, 16). A recent study associated an increase in classical and Toll-like receptor-positive monocytes in early-moderate PD with rapid progression, greater clinical impairment, and early cognitive decline (17). Animal models and clinical studies of PD correlate cytokine dysregulation, specifically, elevated levels of IL-6 and IFN-γ, with the degeneration of dopamine neurons (16). Genomic studies associate PD with mutations/variants of *LRRK2, MHC Class II* (18), and *PINK1* (19, 20), genes implicated in immune cell function.

Studies show that the adaptive immune system is essential to PD pathogenesis (3, 21–24). Two major classes of lymphocytes, T-cells and B-cells, induce adaptive immunity, conferring antigen specificity and immunological memory (16). Patients with PD have elevated numbers of CD4^+^ and CD8^+^ T-cells in the ventral midbrain compared to healthy controls (9). T-cells from PD patients initiate an immune response to post-translationally modified α-Syn, implicating an autoimmune reaction to intrinsic antigens (15, 21). Moreover, a recent study associated α-Syn-specific T-cell reactivity with preclinical and early PD, and pro-inflammatory CD4^+^ T-cells are most abundant shortly after diagnosis of motor symptoms (13).

The JAK/STAT signaling pathway, in which Janus Kinases (JAKs) and Signal Transducers and Activators of Transcription (STATs) proteins interact with cytokine receptors, plays a critical role in the activation and regulation of immune responses (25, 26). Its dysregulation (i.e., over-activation) is associated with many pathological conditions, including multiple sclerosis (MS), rheumatoid arthritis, inflammatory bowel disease, and many cancers (27, 28). Emerging evidence indicates that it polarizes myeloid cells and T-cells to pathogenic phenotypes (29). We previously demonstrated that inhibiting the JAK/STAT pathway ameliorates disease severity in several preclinical models of MS (30) and reduces neuroinflammation in a PD model in which adeno-associated virus (AAV) human α-Syn mediates overexpression of α-Syn (31). However, virally induced α-Syn overexpression can cause up to a 30-fold increase in α-Syn levels and thus does not model idiopathic PD. A pre-clinical model that better mimics PD neuroinflammatory processes is needed to define how inhibiting the JAK/STAT pathway mediates protection against neuroinflammation.

The Thy1-α-Syn transgenic mouse, or Line 61, uses the Thy1 promoter to express full-length, wild-type, human synuclein. It reproduces many features of sporadic PD, including progressive changes in dopamine release in striatal content, α-Syn pathology, motor and nonmotor deficits, neuroinflammation, and biochemical and molecular changes similar to those observed in PD (32). However, the mice do not develop these phenotypes until they are 9–18 months old (32, 33). In another PD model, small, fibrillar seeds of mouse α-Syn induce wild-type mice to form inclusions that biochemically and morphologically resemble those found in PD brains. However, while microglia are activated, this preformed fibril (PFF) model shows minimal expression of the MHC Class II-positive cells that characterize PD (34, 35).

Hypothesizing that introducing human PFFs into mice expressing human α-Syn would better replicate human PD, we injected PFFs generated from purified, recombinant human α-Syn into Line 61 mice (Line 61-PFF). Injection of hu-α-Syn PFFs produced α-Syn inclusions in the SNpc and striatum, induced MHC Class II expression and enhanced innate and adaptive immune cell infiltration and activation in the midbrain, in contrast to injection of monomeric hu-α-Syn.

The Line 61-PFF model enables us to evaluate the contribution of the JAK/STAT pathway to neuroinflammatory responses, and the impact of inhibiting this pathway. Administering the JAK1/2 inhibitor AZD1480 suppressed neuroinflammation and reduced p-α-Syn inclusions. Single-cell RNA Sequencing (scRNA-Seq) revealed that PFF injection induced a specific monocyte/macrophage (MM) cluster expressing antigen-presentation genes and a T-cell cluster expressing numerous proinflammatory genes which correlate with neuroinflammation. Inhibiting the JAK/STAT pathway abrogated the presence of these two PFF-induced immune cell clusters. These findings elucidate neuroinflammatory mechanisms and may inform the development of more specific therapeutic approaches and/or diagnostic biomarkers for patients with PD.

## MATERIALS AND METHODS

### Mice.

The Thy1-α-Syn (Line 61) mouse overexpresses full-length, human, wild-type α-Syn under the Thy-1 promoter. The model is bred by crossing female Line 61 mice with male hybrid B6D2F1 (BDF1) mice and maintained on a congenic background (32). Since the transgene is inserted in the X chromosome, and random inactivation of the X chromosome carrying the mutation in female mice may occur (36), experiments used only littermate males with a Thy1-α-Syn-positive transgene. The University of Alabama at Birmingham (UAB) Institutional Animal Care and Use Committee approved all animal research protocols.

### Human α-Syn Monomer and PFFs.

Human monomeric α-Syn (monomer) was purified using size-exclusion chromatography followed by anion exchange. The Pierce high-capacity endotoxin kit was used to remove endotoxins to < 0.1 EU/μg. α-Syn PFFs were generated as described (37). Monomer concentration was measured using A280 and the extinction coefficient of 5960 M^−1^cm^−1^. Monomer was diluted to 5 mg/ml in 150 mM KCl and 50 mM Tris-HCl buffer and shaken for seven days to generate PFFs. After seven days, PFF protein concentration was determined and previously noted buffer was used to bring concentration to 5 mg/ml. On the day of stereotaxic injections, PFFs were sonicated using a cup horn sonicator (QSonica) with a 15°C water bath. Dynamic light scattering (Wyatt Technology) confirmed < 50 nm fragmentation of PFFs. Immediately before injection, 5 mg/ml of monomer was spun at 20,000 × *g*, and only the supernatant was injected to prevent aggregate formation.

### Intracranial Stereotaxic Injections and Treatment with the JAK1/JAK2 Inhibitor AZD1480.

Male Line 61 mice between the age of 12–15 weeks were anesthetized using isoflurane and received stereotaxic, unilateral, intrastriatal injections using the coordinates relative to bregma: 1 mm A/P, 2 mm M/L, and −3.2 mm D/V relative to the skull (37). Those used in the flow cytometry, scRNA-Seq, and phospho-STAT experiments received bilateral intrastriatal injections for immune cell collection. Two microliters of sonicated PFFs (5 mg/ml) or monomeric α-Syn (5 mg/ml) were injected at 0.5 μl/min with a Hamilton syringe. Mice received 10 units of 1.5 mg/ml carprofen subcutaneously at the time of surgery and the following day.

Two weeks after PFF injections, Line 61-PFF mice were administered either AZD1480 by oral gavage at 25 mg/kg or 0.1% DMSO (Sigma-Aldrich) as a vehicle control every day. The mice were sacrificed two or four weeks later (four- or six-weeks post injection [wpi]), depending on the analysis performed. For examination of TH positive neurons, the mice were sacrificed at a 12-week time point. At time of sacrifice, they were transcardially perfused with cold PBS (pH 7.4), followed by 4% paraformaldehyde for immunofluorescence or cold PBS alone for other experiments.

### Immunofluorescence and Immunohistochemistry.

Prior to sectioning, brains were postfixed in 4% PFA overnight at 4°C, followed by cryoprotection in 30% sucrose/PBS until tissues sank to the bottom. Whole brains were frozen in N-methylbutane at −50°C. Coronal sections 40-μm thick representing the entire brain were serially collected and stored in 50% glycerol in 0.01% sodium azide in Tris-buffered saline (TBS) at −20°C.

Immunofluorescence analysis of free-floating brain sections was performed as previously detailed (31). Sections were then labeled with anti-MHC Class II [A-I/A-E] (clone M5/114.15.2, Invitrogen, cat #14-5321-85), anti-phospho-α-Synuclein (p-α-Syn) (phospho-Serine129, clone EP1536Y, Abcam, cat #ab51253), anti-tyrosine hydroxylase (TH, abcam, cat #ab76442), or anti-IBA1 (polyclonal, Wako, cat #019-197111). After overnight incubation with primary antibodies, sections were washed and incubated with the appropriate Alexa-fluor-conjugated secondary antibodies (Life Technologies). Sections were mounted onto positively charged glass slides and cover-slipped using Prolong Gold (ThermoFisher). Images were acquired using either a Leica TCS-SP5 laser scanning confocal microscope or a Nikon Ti2-C2 confocal microscope and processed using Adobe Photoshop and Illustrator (37).

Free-floating immunohistochemistry was performed as detailed previously (38). Brain sections were labeled with anti-tyrosine hydroxylase (Millipore, ab152), a marker of dopaminergic neurons in mouse midbrain. On day two the sections were labeled with a biotinylated secondary followed by HRP-conjugated amplification complex. DAB chromogenic substrate deposition was timed and visualized by eye (Vector, SK-4100) before mounting on positively charged slides, which were dehydrated and cover-slipped using Permount (Fisher, SP15-500).

### Unbiased Stereology.

To quantify TH neurons in the SNpc, unbiased stereology was performed as described (38). A reviewer blinded to condition coded and analyzed 5–6 TH-DAB-stained slides encompassing the rostro caudal SNpc using an Olympus BX51 microscope and the optical fractionator probe in the StereoInvestigator software (MBF Bioscience). Both ipsilaterally injected and contralaterally uninjected sides of the SNpc were quantified. TH^+^ neurons within the SNpc contours on a 100 μm × 100 μm grid were counted at an optical dissector height of 22 μm. Weighted section thickness was used to account for variations in tissue thickness. Brightfield images of TH^+^ neurons in the SNpc were acquired using the Olympus BX51 microscope.

### Phospho-Synuclein Aggregate Quantitation.

After immunofluorescence staining with anti-α-phospho-Serine129 (p-α-Syn) as described (37, 39), 20X tiled, ipsilateral SNpc confocal images were obtained using the Nikon Ti2-C2 microscope. An ImageJ cell counter was used to count each p-α-Syn-positive neurite and inclusion in the SNpc, which was delineated by drawing a contour using TH^+^ neurons as a reference and then splitting the channels so only p-α-Syn channel fluorescence remained. Two to six SNpc sections were analyzed per animal, depending on their availability and quality after staining.

### Mononuclear Cell Isolation.

Six weeks after bilateral injection of monomer or PFFs, mononuclear cells in the ventral midbrain were isolated as described (31, 40). Midbrain tissue was passed through a 100-μm filter to obtain a single-cell suspension, and mononuclear cells were isolated using a 30/70% Percoll gradient.

### Flow Cytometry.

To detect surface proteins, mononuclear cells were incubated with Fc Block (Bio X Cell, 2.4G2) for 15 min and washed, followed by incubation with viability dye. The indicated antibodies were fluorescently conjugated against CD45 (clone 30-F11, BD Horizon, cat #563410), CD11b (clone M1/70, BD Biosciences, cat #563553), CD11c (clone HL3, BD Pharmingen, cat #553801), CD4 (clone GK1.5, BioLegend, cat #100428), CD8α (clone 53 – 6.7, BioLegend, cat #100734), CD19 (clone 6D5, BioLegend, cat #115541), and MHC Class II (clone M5/114.15.2, BioLegend, cat #107628). Samples were run on a BD Symphony (BD Biosciences) and analyzed using FlowJo software (Tree Star), as described (31, 40, 41).

### Immunoblotting.

Thirty μg of midbrain mononuclear cell homogenate was separated by electrophoresis and probed with antibodies as described (30). All immunoblots represent three individual experiments.

### Single-cell RNA Sequencing (scRNA-Seq).

Mononuclear cells were isolated for sequencing as described above with 3–5 ventral midbrains pooled per sample and sorted for CD45^+^ live cells on a BD FACsAria. Sorted cells were loaded onto the 10X Chromium platform (10X Genomics), and libraries constructed using the Single Cell 3′ Reagent Kit V3.1 according to the manufacturer’s instructions. At least three biological replicates for each group were processed separately (PBS [n = 3], monomer [n = 3], PFF + vehicle [n = 4], PFF + AZD1480 [n = 5]). Samples were sequenced at an average depth of 20,000 reads per cell using Illumina NextSeq 500. Raw base call files were demultiplexed into FASTQ files. Sequencing files were processed and mapped to mm10, and count matrices were extracted using the Cell Ranger Single Cell Software (v 7.1.0) (42).

### scRNA-Seq Analysis.

The count matrices in the h5 file format were imported into the Partek Flow (Partek, Inc.) pipeline (43). Single-cell quality control was performed by applying an inclusion filter on counts per cell (500–15000) and detected genes per cell (250–5000). Cells with more than 10% mitochondrial gene expression were excluded to eliminate apoptotic or dying cells (44). The noise-reduction filter was also applied to exclude features where the value ≤ 0 is in at least 99.9% of cells. The filtered dataset was normalized and scaled with SCTransform workflow.

Principal component analysis (PCA) was performed on the SC-scaled data to graph clustering based on the Louvain algorithm, with the PCA number set to 20. The data were visualized by 3D uniform manifold approximation and projection (UMAP) dimensional reduction using the first 20 principal components. Cell annotations for each cluster were determined using the top differentially expressed genes (DEGs) in computed biomarkers and canonical markers following the classification workflow in Partek Flow (45). Differences in gene expression among samples were determined by the Hurdle model on log2 normalized counts. Dot and violin plots were generated with sc.pl.dotplot and sc.pl.violin functions in Scanpy (1.9.1) (46) using annotated h5ad files exported from Partek workflow.

The MM1 and MM2 clusters were subsets of the original annotated data node in Partek. They were reclustered as MM1 to MM5 following standard PCA and graph-based processes on SCTransform-scaled data and visualized with UMAP. The T-cell cluster was a subset of the original annotated data node in Partek and reclustered as T1 to T5 (47).

### Pathway Enrichment Analysis.

GSEA (gene set enrichment analysis): DEG between individual MG, MM, or T clusters was determined via the Partek gene-specific analysis (GSA) test. The exported DEG list was ranked by −log(P) and converted to an RNK file uploaded to GSEA software (version 4.3.2, BROAD Institute). GSEAPreRanked chooses a hallmark gene-set database (48). Pathway analysis results were plotted in terms of a normalized enrichment score (NES) and a false discovery rate (FDR) using the ggplot2 (version 3.4.0) package in RStudio.

### Statistical Analysis.

Flow cytometry experiments used 3–5 independent samples per group, with two ventral midbrains per sample (6–10 mice per experiment). Data were analyzed using an unpaired t-test (two-tailed) or two-way ANOVA with Tukey’s multiple comparison test with nested design. Graphs display the individual values and mean ± SEM, with *p < 0.05, **p < 0.01, ***p < 0.001, and ****p < 0.0001.

### Data Set Availability.

ScRNA-Seq data will be available online. The single-cell data have been deposited in the GEO under the accession number GSE264525. Raw files supporting our findings are available from the corresponding authors upon reasonable request.

## RESULTS

### Novel Pre-clinical PD Model (Line 61-PFF).

**Figure 1:**
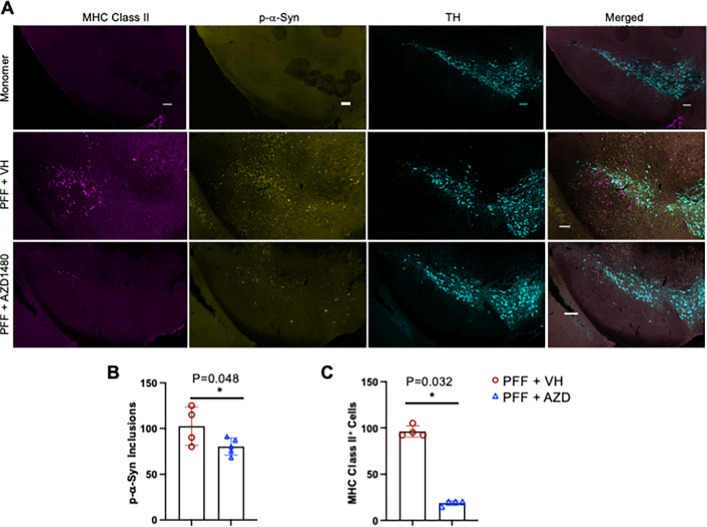
Increased MHC Class II Expression and a-Syn Phosphorylation in Line 61-PFF Mice. Male Line 61 mice (12–15 weeks old) were injected in the striatum with human a-Syn monomer (10 mg) or hPFF (PFF) (10 mg). Four weeks later, those injected with human a-Syn monomer were perfused for immunofluorescence. Two weeks later, those injected with hPFF, were treated with VH (DMSO) or AZD1480 (25 mg/kg/day) for an additional two weeks for a total of four weeks. **A.** Immunofluorescence performed in the SNpc detected MHC Class II (magenta), phospho-a-Syn (p-a-Syn) (yellow), or TH (cyan). The abundance of MHC Class II-positive cells or p-a-Syn inclusions was quantified using Fiji. **B.** An independent t-test showed significant differences in the number of MHC Class II-positive cells between VH- and AZD1480-treated groups. **C.** An independent t-test showed significant differences in the number of p-a-Syn-positive aggregates between groups. Scale bar = 100 μm. *p < 0.05.

We previously showed that the JAK1/2 inhibitor AZD1480 prevents neuroinflammation and protects against dopaminergic neuron loss in response to AAV-human-α-Syn overexpression in rats (31). To better mimic idiopathic PD, we tested mouse PFFs and found that injection into mouse striatum activated microglia but did not induce the accumulation of MHC Class II-expressing immune cells (data not shown) that occur in human PD brains (49). We therefore tested whether injecting *human* α-Syn PFFs into the striatum of Line 61 mice, which use the Thy1 promoter to express human α-Syn (32), would induce more robust inflammatory phenotypes. Using an antibody to α-Syn phosphorylated at serine 129 (p-α-Syn), we identified α-Syn inclusions in the SNpc four weeks after injecting Line 61 mice with human PFFs (Figs. 1A, B). We also observed abundant MHC Class II-positive immune cells in the SNpc (Figs. 1A, 1C). Injecting monomeric α-Syn into Line 61 mice did not produce p-α-Syn inclusions or MHC Class II-positive cells (Fig. 1A).

We tested the JAK1/2 inhibitor AZD1480 in the Line 61-PFF model as described (31). Two weeks after PFF injections, AZD1480 or VH was administered for two weeks, then mice were sacrificed for immunofluorescence studies. Figures 1A–C show that AZD1480 treatment significantly reduced α-Syn inclusions and the number of MHC Class II-positive cells in the SNpc.

Sections from the SNpc were stained for TH to determine the number of dopaminergic neurons in mice injected with monomer or PFFs and treated with VH or AZD1480. Line 61-PFF mice did not exhibit a loss of TH-positive neurons compared to monomer-injected mice at a 12-week time point, and AZD1480 treatment produced no significant differences (Supplemental Figs. 1A, B).

### Neuroinflammation in Line 61-PFF Mice.

**Figure 2:**
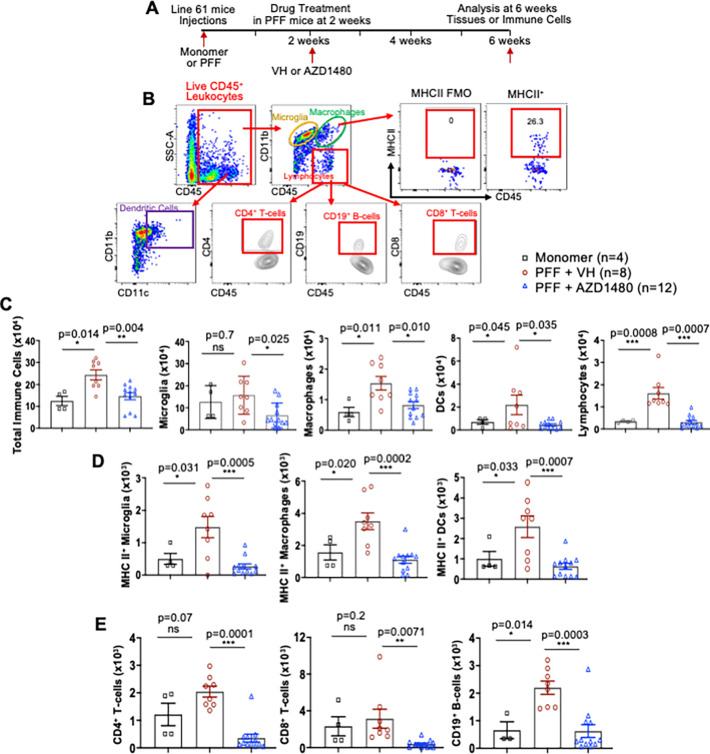
Experimental Design, Gating Strategy, and Flow Analysis of Innate and Adaptive Immune Cells from the Midbrain. **A.** Male Line 61 mice (12–15 weeks old) were injected in the striatum with monomer (10 mg) or PFF (10 mg). Two weeks after PFF injection, VH (DMSO) or AZD1480 (25 mg/kg/day) was administered by oral gavage for an additional four weeks for a total of six weeks. **B.** Mononuclear cells were isolated from midbrains. The gating strategy of flow cytometry analysis was as follows: CD45^Med^CD11b^+^ microglia, CD45^Hi^CD11b^+^ macrophages, CD45^+^CD11b^+^CD11c^+^ dendritic cells (DCs), CD45^+^CD11b^−^CD4^+^ T-cells, CD45^+^CD11b^−^CD8^+^ T-cells, and CD45^+^CD11b^−^CD19^+^ B-cells. Representative gating plots of MHC Class II expression in microglia, macrophages, and DCs are shown. Fluorescence minus one (FMO) was used as a control. **C.** Six weeks post injection, mononuclear cells were isolated from the midbrains of monomer (n=4), PFF + VH (n=8), or PFF + AZD1480 (25 mg/kg/day) (n=12) mice, then subjected to flow cytometry analysis. Absolute numbers of total CD45^+^ immune cells, CD45^Med^CD11b^+^ microglia, CD45^Hi^CD11b^+^ macrophages, CD45^+^CD11b^+^CD11c^+^ DCs, and CD45^+^CD11b^−^ lymphocytes are shown as mean ± SD. **D.** Absolute numbers of MHC Class II-positive CD45^Med^CD11b^+^ microglia, macrophages, and DCs are shown as mean ± SD. **E.** Absolute numbers of CD4^+^ T-cells, CD8^+^ T-cells, and CD19^+^ B-cells are shown as mean ± SD. Statistical significance was determined by ordinary one-way ANOVA. *p < 0.05, **p < 0.01, ***p < 0.001.

Injecting human PFFs into the striatum of Line 61 mice increased MHC Class II positive immune cells in the SNpc, and AZD1480 treatment reduced the abundance of these cells (Fig. 1). Immune cell phenotypes in the midbrain were further characterized by flow cytometry (Figs. 2A, B). In Line 61-PFF mice, total immune cells in the midbrain were significantly higher than in monomer α-Syn-injected mice (Fig. 2C). We identified microglia as CD45^Mid^CD11b^+^, macrophages as CD45^Hi^CD11b^+^, dendritic cells (DCs) as CD45^Hi^CD11b^+^CD11c^+^, and lymphocytes as CD45^+^CD11b^−^. In Line 61-PFF mice, macrophages, DCs, and lymphocytes showed significant increases compared to monomer-injected mice, but the total number of microglia did not increase (Fig. 2C).

We assessed the activation status of innate immune cell subsets by determination of MHC Class II expression. Absolute numbers of MHC Class II positive microglia, macrophages, and DCs were significantly higher in the midbrain of Line 61-PFF mice than in the monomer group (Fig. 2D).

Since adaptive immune cells are also implicated in PD (3, 21–24), we determined how pathologic α-Syn affects infiltration of CD4^+^ T-cells, CD8^+^ T-cells, and CD19^+^ B-cells. We found a significant increase in CD19^+^ B-cell but not CD4^+^ or CD8^+^ T-cell infiltration in Line 61-PFF mice compared to the monomer group, although CD4^+^ T-cell infiltration trended towards significance (Fig. 2E).

We next determined how AZD1480 treatment influenced specific subsets of immune cells (Figs. 2A, B). AZD1480 or VH was administered two weeks after PFF injections, and treatment continued for four weeks, when mice were sacrificed for analysis. Compared to VH, AZD1480 significantly suppressed absolute numbers of immune cells (microglia, macrophages, DCs, and lymphocytes), the numbers of MHC Class II positive microglia, macrophages and DCs, and the number of CD4^+^ T-cells, CD8^+^ T-cells, and CD19^+^ B-cells in the midbrain of Line 61-PFF mice (Figs. 2C–E). These data show that inhibiting the JAK/STAT pathway suppresses the infiltration and activation of innate and adaptive immune cells.

### PFF Induction of STAT3 Activation.

**Figure 3:**
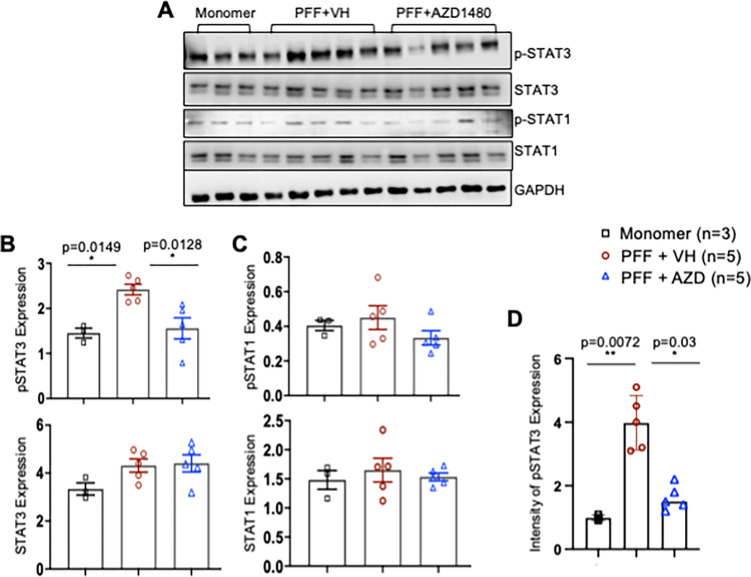
AZD1480 Inhibits PFF-induced STAT3 Phosphorylation in the Midbrain. **A.** Male Line 61 mice (12–15 weeks old) were injected in the striatum with monomer (10 mg) or PFF (10 mg). Two weeks after PFF injection, VH or AZD1480 (25 mg/kg/day) was administered by oral gavage for an additional four weeks. Lysates were obtained from mononuclear cells isolated from the midbrains of monomer (n=3), PFF + VH (n=5), or PFF + AZD1480 (n=5) mice at six weeks and immunoblotted with the indicated antibodies. **B.** Fold-induction of p-STAT3 and total STAT3 was calculated by normalizing to GAPDH using ImageJ 1.53t. **C.** Fold-induction of p-STAT1 and total STAT1 was calculated by normalizing to GAPDH using ImageJ 1.53t. **D.** Quantification of p-STAT3 levels in the SNpc (4 sections/sample) using immunohistochemistry from monomer (n=3), PFF + VH (n=5), or PFF + AZD1480 (n=5) mice six weeks post injection. Statistical significance was determined by ordinary one-way ANOVA. *p < 0.05, **p < 0.01.

Activation of the JAK/STAT pathway results in phosphorylation of STAT proteins (28). We assessed activation in Line 61-PFF mice by measuring tyrosine phosphorylation of STATs, particularly STAT1 and STAT3 (30, 50). Immunoblotting was performed to measure protein expression levels of total STAT1, phosphorylated STAT-1 (p-STAT1), total STAT3, and p-STAT3 in mononuclear cells isolated from the midbrain. We observed a significant increase in p-STAT3 in Line 61-PFF mice treated with VH compared to monomer, which was inhibited by AZD1480 treatment (Figs. 3A, B). There were no statistical differences in total STAT3, total STAT1, and p-STAT1 expression (Figs. 3A–C). Immunohistochemistry confirmed the significant increase in p-STAT3^+^ cells in Line 61-PFF mice treated with VH compared to monomer treatment, and suppression after AZD1480 treatment (Fig. 3D). These data indicate that PFF injection activates the JAK/STAT pathway as demonstrated by phosphorylated STAT3, and AZD1480 inhibits STAT3 activation in mononuclear cells.

### scRNA-Seq Characterization of Immune Cell Clusters in Line 61-PFF Mice.

Several scRNA-Seq studies point to the heterogeneity of cells involved in PD pathogenesis (51–54). To identify cell-specific contributions in the Line 61-PFF model, we performed scRNA-Seq on sorted CD45^+^ leukocytes obtained from Line 61 mice injected with PBS, monomer, PFF plus VH, or PFF plus AZD1480 (Supplemental Fig. 2). Cell clusters were annotated with the top differentially expressed biomarkers and canonical markers for microglia (MG), monocytes/macrophages (MM), T-cells (T), B-cells (B) and neutrophils (Neu) (Supplemental Fig. 3A). We observed no difference in the percentage of each cluster or total cell numbers between the PBS and monomer groups, indicating that monomer injection did not affect the immune cell subsets found in PBS-treated Line 61 mice (Supplemental Figs. 3B, C). Most cells (> 80%) were identified as MG and separated into 9 clusters (MG1-9; Supplemental Fig. 3A). Upon PFF injection, either the percentage or absolute numbers of MG2, MG4, and MG5 clusters were higher than those in PBS- and monomer-injected mice (Supplemental Figs. 3B, C). Percentages and cell numbers of MM clusters and the T-cell cluster were higher in PFF injected mice than in PBS- and monomer-injected mice (Supplemental Figs. 3B, C). Neu and B-cell clusters were not examined due to low (< 1%) numbers.

### scRNA-Seq Reveals an PFF-induced MM Inflammatory Cluster Which is Modulated by AZD1480 Treatment.

**Figure 4:**
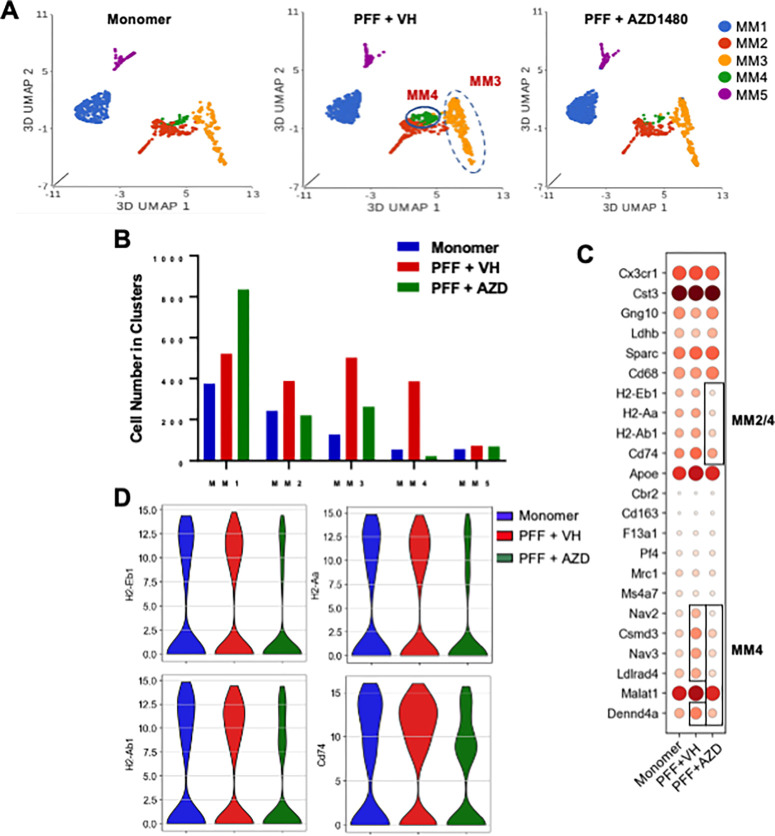
scRNA-Seq Reveals an PFF-induced MM Inflammatory Cluster Which is Modulated by AZD1480 Treatment. **A.** UMAP visualization of MM clusters from monomer, PFF + VH, or PFF + AZD1480 (25 mg/kg/day) mice. **B.** Cell numbers of the five MM clusters analyzed by scRNA-Seq. **C.** Dot plot of conserved marker genes in the five MM clusters across groups. **D.** Violin plots showing the expression-level distribution of MHC Class II genes across groups.

We found no differences in MM cell cluster percentage or numbers between the PBS and monomer groups (Supplemental Figs. 3B, C). To further characterize MM clusters, we used the original annotated dataset to recluster MM1 and MM2 as MM1-MM5 (Supplemental Fig. 3D). MM1, MM2, and MM3 were the major clusters identified in the monomer group (Figs. 4A, B). Examining their transcriptional profiles, *Gng10* and *Ldhb* were highly expressed in the MM1 cluster, while genes related to antigen presentation, *H2-Eb1, H2-Aa, H2-Ab1,* and *Cd74* (55, 56), were enriched in MM2 and MM4 (Supplemental Figs. 4A, B). Genes related to macrophage function and signal transduction, including *F13a1, Cd163, Mrc1, Ms4a7,* and *Pf4* (57) were differentially expressed in MM3, while *Nav2, Nav3, Ldlrad4, Dennd4a,* and *Csmd3*, genes associated with Alzheimer’s Disease, Autism Spectrum Disorders (ASD), aging, axon guidance and signal transduction (58–63) were elevated in MM4. Only MM5 expressed *Nfib, Dnm3,* and *Fermt2* (Supplemental Figs. 4A, B). GSEA revealed that the MM2 and MM4 clusters were enriched in IFN-α and IFN-γ responses, IL-6-JAK-STAT3 signaling, TNF-α signaling via NF-κB, Hedgehog signaling, inflammatory responses and hypoxia (Supplemental Fig. 4C).

We examined whether the MM clusters changed with PFF injection. MM1, MM2, MM3, and MM4 cell numbers increased in Line 61-PFF mice compared to monomer, with the MM4 cluster showing the greatest increase (Figs. 4A, B), and DEG analysis revealed upregulated expression of MM4 genes (Fig. 4C).

AZD1480 treatment reduced MM2, MM3, and MM4 cell numbers, almost eliminating the MM4 cluster, and increased MM1 cell numbers (Fig. 4B). *H2-Eb1, H2-Aa, H2-Ab1,* and *Cd74* genes were suppressed in the MM2 and MM4 clusters (Figs. 4C, D) and *Nav2, Csmd3, Nav3, Ldlrad4,* and *Dennd4a* in the MM4 cluster (Fig. 4C). Collectively, these results suggest that MM4 is a potentially pathogenic cluster that emerges after PFF injection, and AZD1480 treatment abrogates the appearance of the MM4 cluster.

### AZD1480 Treatment Reduces MG Cluster Cell Numbers but Does Not Influence Transcriptional Profiles in Line 61-PFF Mice.

Microglia play a critical role in PD pathogenesis (64–66), so we assessed their transcriptional profiles in Line 61 mice. MG1, MG3, MG4, and MG6 were the major MG clusters found in the PBS and monomer groups, with no differences in the percentage or cell numbers of the 9 MG clusters (Supplemental Figs. 3B, C). All MG clusters expressed the canonical gene markers *P2ry12* and *Cx3cr1*. MG1 strongly expressed *Klf2* and *Egr3*, MG3 expressed *Maf* and *Slc2a5*, and MG5 expressed *Spp1* and *Apoe*. Although MG2 primarily upregulated *Gm42418* and *Cmss1,* and MG4, *Gng10* and *Alox5ap*, neither does so exclusively. MG6 upregulated two genes, *Snx29* and *Anks1*, and downregulated *Maf* and *Slc2a5*. The MG7 cluster upregulated *Stat3* and *Cd83* and downregulated *Klf2* and *Egr3*. MG8 expressed *Top2a* and *Mki67*, and MG9 uniquely expressed *Ifit2*, *Ifit3*, *Oasl*, and *Irf7* (Supplemental Figs. 5A, B). GSEA revealed that the MG2 and MG9 clusters were enriched in IFN-α and IFN-γ response pathways, and MG2 was also enriched in TGF-β and PI3K-AKT-MTOR signaling (Supplemental Fig. 5C).

**Figure 5:**
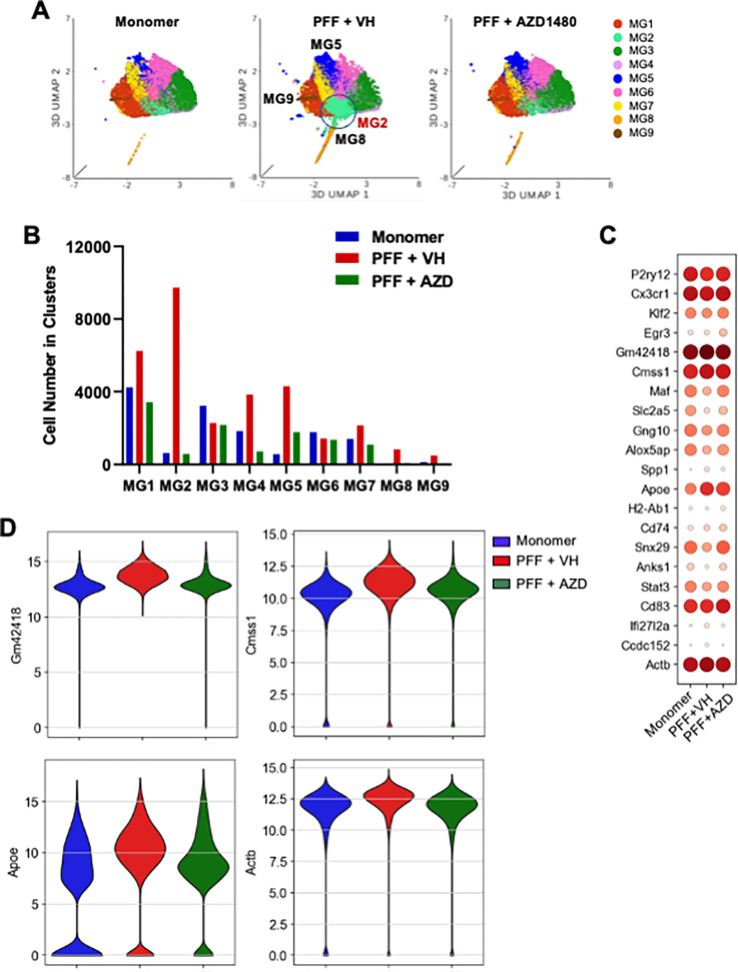
AZD1480 Treatment Reduces Cell Numbers but Does Not Influence MG Clusters’ Transcriptional Profiles in Line 61-PFF Mice. **A.** UMAP visualization of MG clusters from monomer, PFF + VH, or PFF + AZD1480 (25 mg/kg/day) mice. **B.** Cell numbers of the nine MG clusters analyzed by scRNA-Seq. **C.** Dot plot of conserved marker genes in the nine MG clusters across groups. **D.** Violin plots showing the expression-level distribution of genes related to inflammatory responses across groups.

PFF injection increased MG2, MG4, MG5, MG8, and MG9 cell numbers compared to monomer injection (Figs. 5A, 5B), with MG2 showing the greatest increase. However, PFF injection did not change transcriptional profiles to any great extent (Fig. 5C). While AZD1480 treatment reduced cell numbers of MG1, MG4, MG5, MG8, and MG9 clusters overall and MG2 cell numbers to the low levels seen in monomer mice (Fig. 5B), DEG analysis identified only subtle changes in *Gm42418, Cmss1, Apoe,* and *Actb* (Figs. 5C, D).

### scRNA-Seq Reveals an PFF-induced T-cell Inflammatory Cluster Which is Modulated by AZD1480 Treatment.

**Figure 6:**
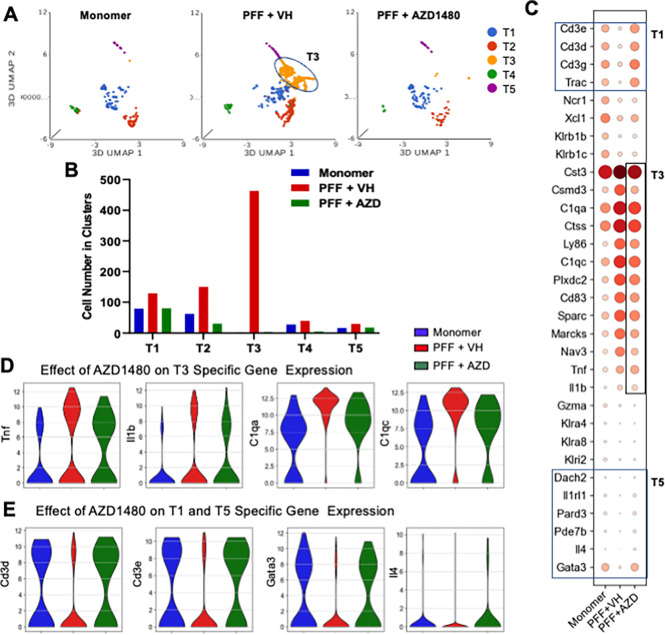
T-cell Clusters Associated with PFF-induced Neuroinflammation and the Influence of AZD1480 Treatment. **A**. Unsupervised clustering of scRNA-Seq data and UMAP plot of all T-cells (T) from CD45^+^ mononuclear cells sorted from the midbrain of Line 61 mice after monomer, PFF + VH, or PFF + AZD1480 injection. **B.** Cell numbers of the five T-cell clusters analyzed by scRNA-Seq. **C.** Dot plot of conserved marker genes in the five T-cell clusters across groups. **D.** Violin plots show the genes restored by AZD1480 treatment in the T3 cluster across groups. **E.** Violin plots showing genes restored by AZD1480 treatment in the T1 and T5 clusters across groups.

We identified five T-cell clusters (T1 to T5; Fig. 6A and Supplemental Figs. 3A, E). Most of the T-cells in monomer-treated mice were in cluster T1, which strongly expressed *Cd3e, Cd3d, Cd3g,* and *Trac*, genes encoding TCR (67). The T2 cluster expressed *Ncr1, Xcl1, Klrb1b,* and *Klrb1c; T4, Gzma* and *Klri2;* and T5, *Gata3*, a gene related to Th2 differentiation (68, 69) (Supplemental Figs, 6A, B). Strikingly, PFF injection uniquely induced a new cluster, T3, which most strongly expressed numerous proinflammatory genes, including *Cst3, Csmd3, C1qc, C1qa, Cd83, Tnf,* and *Il1b* (Figs. 6A, B; Supplemental Figs. 6A-C). GSEA revealed that the T2 and T4 clusters were enriched in the IFN-γ response pathway and IL-2-STAT5 pathway, while PI3K-AKT-MOTR, IL-6-JAK-STAT3, TNF-α signaling via NF-κB and Notch signaling were enhanced in the T5 cluster. The T3 cluster showed enriched Myc-target signaling (Supplemental Fig. 6E). Cell numbers in the T1 and T2 clusters were increased by PFF injection, and as mentioned previously, the T3 cluster emerged only after PFF injection (Fig. 6B).

AZD1480 treatment reduced T1 and T2 cell numbers, with an almost complete abrogation of the T3 cluster (Figs. 6A, B). *Tnf, Il1b, C1qa*, and *C1qc* gene expression was also reduced upon AZD1480 treatment (Figs. 6C, D). Interestingly, AZD1480 restored expression of the naïve T-cell-related genes *Cd3d* and *Cd3e* (70, 71) in the T1 cluster as well as the Th2-related genes *Gata3* and *Il4* (72) in the T5 cluster (Figs. 6C, E).

Collectively, these results reveal that a novel inflammatory T-cell cluster, T3, is induced in response to PFF injection, and is associated with neuroinflammatory responses. AZD1480 treatment significantly decreased T3 cell numbers and suppressed proinflammatory gene expression. AZD1480 also restored T1 and T5 transcriptional profiles, suggesting that the JAK/STAT pathway affects T-cells in the Line 61-PFF model of PD.

## DISCUSSION

Developing a pre-clinical model of PD that closely resembles human PD is crucial in understanding the mechanisms that influence onset and progression of the disease and identifying potential therapeutic targets. We have generated a new pre-clinical PD model by injecting human PFF into Line 61 mice that express wild-type human α-Syn. This model, Line 61-PFF, is characterized by increased expression of MHC Class II and infiltration and activation of innate and adaptive immune cells in the midbrain, thereby exhibiting a strong neuroinflammatory response. We utilized single-cell transcriptomics, a powerful research tool that enables high-resolution analysis of gene expression (45, 73–75) to evaluate the transcriptional profiles of immune cells infiltrating into the midbrain of Line 61-PFF. Most strikingly, we identified two unique clusters, MM4 and T3, that were induced by PFF injection. A JAK1/2 inhibitor, AZD1480, was used to investigate the effect of blocking the JAK/STAT pathway on neuroinflammation. Our results revealed an immunosuppressive effect of JAK1/2 inhibition by reducing immune cell infiltration in the brain, and inhibiting proinflammatory transcriptional profiles. These findings indicate the therapeutic potential of JAK/STAT blockade in the treatment of neurodegenerative diseases with pronounced neuroinflammation.

To define the heterogeneity of immune cells involved in neuroinflammation in Line 61-PFF mice, scRNA-Seq was utilized. We identified 5 monocyte/macrophage (MM) clusters and a significant effect of PFF injection on the MM4 cluster, enhancing expression of the antigen-presentation genes *H2Eb1, H2-Aa, H2-Ab1*, and *Cd74* as well as genes related to neurological diseases and pro-inflammatory M1 polarization, including *Nav2, Nav3, Malat1, Csmd3, Ldlrad4*, and *Dennd4a* (58–63). Neuron navigator 2 (*Nav2*) is highly expressed in patients with rheumatoid arthritis (RA); it is considered a pathogenic gene for RA (76), and STAT3 activation has been shown to upregulate its expression in RA synoviocytes (77). Several single-nucleotide polymorphisms (SNPs) in the *Nav2* gene are associated with the risk and age at onset of AD (78). Note that STAT3 is activated in Line 61-PFF mice (Fig. 3), and AZD1480 treatment suppresses *Nav2* expression in the MM4 cluster (Fig. 4C). In addition, GSEA revealed that the MM4 cluster was enriched in TNF-α signaling (Supplemental Fig. 4C), and TNF-α has been shown to significantly increase *Nav2* expression (76). In Line 61-PFF mice, we found enriched *Tnf* gene expression in the PFF-induced T3 cluster (Supplemental Fig. 6A), suggesting that the increase in MM4 *Nav2* expression may be induced by T3-derived TNF-α. The expression of CUB and Sushi multiple domains 3 (*Csmd3*) and neuron navigator 3 (*Nav3*), genes related to ASD (58–61), is also elevated in the MM4 cluster and suppressed by AZD1480 treatment (Fig. 4C).

Recent studies demonstrated that monocyte-derived disease inflammatory macrophages (DIMs) accumulate during aging, inflammation, and AD (79–81). The DIM-conserved transcriptional signature includes genes such as *Il1a, Il1b, Tnf, Cd49f, Cd54* and *Cd83* (79). The MM4 cluster induced by PFF injection expresses many of these genes. Enrichment analysis identified that the MM4 cluster was enriched in inflammatory response, ROS signaling, and TNF-α signaling pathways (Supplemental Fig. 4C), indicating a pro-inflammatory phenotype. These observations indicate the MM4 cluster identified in Line 61-PFF mice exhibits inflammatory gene signatures of DIMs (79). Importantly, AZD1480 treatment abrogated the presence of the MM4 cluster. These findings identify the MM4 cluster as a potential therapeutic target for PD.

The MM3 cluster identified in Line 61-PFF mice resemble the disease-activated border-associated macrophages (DaBAMs) identified in another α-Syn model of PD (57). Both are characterized by high expression of genes such as *Cd163, F13a1, Pf4, Mrc1, Ms4a7*, and *Apoe*. ApoE mediates neuroinflammation and neurodegeneration in AD (82, 83). The *APOE* genotype directly influences the development of α-synuclein pathology in PD dementia (84, 85). Mouse models of α-synucleinopathy showed that *APOE4* exacerbated α-synuclein pathology in the absence of amyloid (86, 87). *Apoe* expression was elevated in the MM3 cluster (Supplemental Fig. 4A) and suppressed by AZD1480 treatment (Fig. 4C). Collectively, our results point to MM4 and MM3 as potentially pathogenic clusters and demonstrate that AZD1480 treatment inhibits proinflammatory gene expression to reduce neuroinflammation.

Studies demonstrate the essential role of T-cells in the neuropathogenesis of PD (9, 15, 16, 21) and associate α-Syn-specific, proinflammatory CD4^+^ T-cell reactivity with preclinical and early PD (13). Several scRNA-Seq studies have focused on T-cells isolated from the blood and cerebrospinal fluid of PD patients, demonstrating the differentiation and expansion of peripheral CD8^+^ T-cells and CD4^+^ T-cells, as well as the interaction of peripheral CD4^+^ T-cells with endothelial cells in PD patients (53, 88, 89). A recent snRNA-Seq study revealed an increased frequency of T-cells within the SNpc from postmortem PD patients (52). However, little is known about the transcriptional features of T-cells in PD, especially T-cells infiltrating the CNS. We identified increased percentages and numbers of T-cell clusters in Line 61-PFF mice (Supplemental Fig. 3 and Figs. 6A, B). Wang et al revealed activated and expanded T-cell populations in the blood of PD patients compared to healthy controls (89). The T-cell cluster 11 in their study was enriched in *KLRB1* (kill cell lectin like receptor B1) expression with clonal expansion in the blood. Our scRNA-Seq analysis showed that the T2 cluster had elevated expression of *Klrb1b* and *Klrb1c* (orthologous to human *KLRB1*), which resembles the human T-cell cluster C11 in peripheral blood of patients with PD (89). Another scRNA-Seq study using human PD peripheral blood showed that PD-associated cytotoxic CD4^+^ T-cells exhibited a significant increase in proportion and enhancement of IFN-γ responses (53). Consistent with this finding, GSEA demonstrated that the IFN-γ response pathway was enriched in T2 and T4 clusters from Line 61-PFF mice (Supplemental Fig. 6E).

Our scRNA-Seq analysis demonstrated a novel PFF-induced T-cell cluster, T3, which specifically expressed proinflammatory genes, including *Cst3, Csmd3, C1qc, Tnf,* and *Il1b*. AZD1480 treatment abolished the T3 cluster (Fig. 6B) and, surprisingly, restored the naïve T-cell cluster (T1) as well as expression of the Th2-related genes *Gata3* and *Il-4* in the T5 cluster (Fig. 6E). *Il4* and *Gata3* are critical for the differentiation of CD4^+^ Th2 cells, which have immunosuppressive functions (68, 69). Interestingly, idiopathic PD patients show low Gata3 mRNA levels (90), consistent with another study reporting significantly few Th2 cells in PD patients (91). IL-4 attenuates the inflammatory responsiveness of macrophages, which limits inflammasome activation, IL-1β production, and pyroptosis (92). We surmise that JAK inhibition may promote immunosuppressive functions in adaptive T-cells. However, the exact role of Th2 cells, *Gata3*, and *Il-4* in PD remains largely unknown. The JAK-STAT pathway has a critical role in the fate of T helper cell (Th) differentiation (26, 93). STAT3 plays a crucial role in T-cell pathogenicity, promoting inflammatory responses (94–97). In Line 61-PFF mice, STAT3 is activated in mononuclear cells from the midbrain, which may lead to the promotion of proinflammatory T-cells. AZD1480 treatment reduced STAT3 activation, which was associated with reduced neuroinflammation and abrogation of the T3 cluster.

In Line 61 mice, there is considerable pathology at 9–18 months of age, including accumulation of α-Syn, degeneration of TH positive neurons, microglial and astrocytic activation, elevation of inflammatory markers and CD4^+^ T-cell infiltration into the brain (36, 98). In Line 61-PFF mice, we observed a much earlier demonstration of α-Syn inclusions and neuroinflammatory responses at 4–6 weeks. However, no TH positive neuronal loss was observed in this model at 12 weeks following PFF injection, thus we were not able to assess the impact of inhibiting the JAK/STAT pathway on the neurodegenerative process. Future studies will require examination of longer time points or the use of other models with significant dopamine neuron loss in the SNpc to determine if neurodegeneration can be influenced by JAK/STAT pathway inhibition.

## Conclusion

In summary, studies in the novel Line 61-PFF model, which displays a strong neuroinflammatory response, identified two specific immune cell clusters, MM4 and T3, that express high levels of genes related to antigen presentation and neuroinflammation. We also showed the clinical potential of inhibiting the JAK/STAT pathway critical to immune cell infiltration and expression of signature genes in these pathogenic macrophage and T-cell clusters. Future studies targeting these genes/clusters may inform diagnostic markers and/or therapeutic approaches to prevent or ameliorate currently incurable neuroinflammatory and neurodegenerative diseases, such as PD.

## Data Availability

ScRNA-Seq data will be available online. The single-cell data have been deposited in the GEO under the accession number GSE264525.

## Abbreviations

AAV: adeno-associated virus
a-Syn: a-synuclein
Csmd3: CUB and Sushi multiple domains 3
DaBAMs: disease-activated border-associated macrophages
DCs: dendritic cells
DEGs: differential expressed genes
DIMs: disease inflammatory macrophages
FDR: false discovery rate
GSEA: gene set enrichment analysis
JAK: Janus Kinases
KLRB1: kill cell lection like receptor B1
MG: microglia
MM: monocyte/macrophage
MS: multiple sclerosis
Nav2: neuron navigator 2
Nav3: neuron navigator 3
NES: normalized enrichment score
Neu: Neutrophils
p-a-Syn: phosphor-Syn
PCA: principal component analysis
PD: Parkinson’s disease
PFF: performed fibril
RA: rheumatoid arthritis
scRNA-Seq: single-cell RNA Sequencing
SNpc: substantia nigra pars compacta
snRNA-Seq: single-nucleus RNA Sequencing
STAT: Signal Transducers and Activators of Transcription
TNF: tumor necrosis factor
UMAP: uniform manifold approximation and projection
VH: vehicle
